# Evaluation of transcutaneous bilirubinometer (DRAEGER JM 103) use in Zimbabwean newborn babies

**DOI:** 10.1186/s40748-017-0070-0

**Published:** 2018-01-18

**Authors:** Gwendoline Lilly Tanyaradzwa Chimhini, Simbarashe Chimhuya, Vasco Chikwasha

**Affiliations:** 1 Department of Paediatrics and Child Health, University of Zimbabwe-College of Health Sciences, Mazoe Street, Box A178 Avondale, Harare, Zimbabwe; 2 Department of Community Medicine, University of Zimbabwe-College of Health Sciences, Mazoe Street, Box A178 Avondale, Harare, Zimbabwe

**Keywords:** Zimbabwe, Jaundice, Neonate, Bilirubin, JM-103, Correlation

## Abstract

**Background:**

Acute Bilirubin Encephalopathy in the neonatal period is a major cause of permanent disability. Effective screening and surveillance are essential in the newborn period to enable timely management. Noninvasive transcutaneous bilirubin devices have been successfully used for screening in many settings. We evaluated the accuracy of the Draeger JM 103 (Medical Systems, USA) for estimating serum bilirubin in Zimbabwean newborns.

**Methods:**

Paired transcutaneous (forehead and sternum) and serum bilirubin measurements were compared on 283 infants consecutively recruited between 01 August and 30 November 2015 at Harare Hospital Neonatal Unit. Using serum bilirubin as gold standard, Pearson Correlation Coefficient (r) was calculated for the two transcutaneous measurement sites. Linear regression plots of transcutaneous versus serum estimates were performed. Comparison was made between preterm and term babies. Specificity, sensitivity, positive predictive value and negative predictive value of the JM103 were calculated including ROC curves to assess the accuracy of the diagnostic tests.

**Results:**

Fifty-five percent of the babies were male. Median gestational age was 38 weeks (range 28–42). One hundred and fifteen (41%) were preterm. Median postnatal age was 3 days (range 0–10). Serum bilirubin ranged 85–408 μmol/l, transcutaneous bilirubin sternum; 170–544 μmol/l and forehead; 119–510 μmol/l. Correlation between serum and transcutaneous bilirubin (sternum) was 0.77 and between serum and transcutaneous (forehead) was 0.72. Preterm babies correlation for sternum was 0.77 and forehead was 0.75. Term babies correlation for sternum was 0.76 and forehead was 0.70. The sensitivity for the sternum site was 76%, specificity 90%, Positive Predictive Value of 70 and Negative Predictive Value 92. Sensitivity for forehead site was 62%, specificity 95% with a Positive Predictive Value of 80 and Negative Predictive Value of 90. Bland-Altman plot of serum versus transcutaneous measurements showed agreement between the tests. The ROC curves showed that the accuracy of the two diagnostic tests were good with no significant difference between the two, *p* = 0.2954.

**Conclusion:**

The study demonstrated a strong positive correlation for both sternum and forehead sites with serum bilirubin in this Zimbabwean population of African origin. However, the sternum is a better site for identifying babies with jaundice compared to forehead. The Draeger JM-103 can be used to screening for neonatal jaundice in this population.

## Background

Acute Bilirubin Encephalopathy (ABE), resulting from severe neonatal jaundice, is a cause of permanent disability in children in Zimbabwe. ABE is preventable if diagnosed and treated before serum bilirubin reaches dangerous levels. ABE is a condition that has been virtually eliminated as a cause of cerebral palsy in most developed countries [1].

Effective screening and surveillance are essential to ensure that infants with severe or pathological jaundice are timely identified and correctly managed in the immediate newborn period. Newborn jaundice is known to have cephalo-caudal progression [2]. However visual assessment of jaundice has been shown to correlate poorly with measured serum bilirubin levels [3]. Visual assessment of the skin and sclera, though well documented as inaccurate, is employed where objective means of measurement are unavailable, and is a major reason for inadequate case identification of jaundiced newborns by health workers [3–5]. This is even more difficult in darkly pigmented infants resulting in late referral for care when infants are in an advanced state of bilirubin encephalopathy.

In the majority of babies on our unit phototherapy is initiated empirically upon identification of jaundice before laboratory determination of the actual level of serum bilirubin. In our hospital laboratory determination of total serum bilirubin (TSB) is not always possible due to various health system constraints such as unavailability of testing reagents or machine breakdown. Similar constraints are also experienced at other health institutions in the public sector, particularly in rural areas. Similar problems affect health institutions in most resource limited countries. The majority of hospitals in Zimbabwe, therefore, manage jaundiced infants using visual estimation.

Melanin affects the clinical estimation of jaundice in the newborn [6]. Clinical assessment of jaundice often leads to over or underestimation of jaundice and results in unnecessary blood draws from the baby with resultant maternal anxiety [7, 8]. Blood sampling for the estimation of serum bilirubin is one of the commonest tests ordered in the neonatal units and postnatal wards. This is often done by heel prick and is considered painful [8, 9] with potential long-term consequences [9]. Turnaround time for obtaining the laboratory result varies between 2 and 24 h in our hospital. In the event of stock-out of reagents to measure TSB in the hospital laboratory, most parents cannot afford the 20 USD charged by the private laboratories.

Transcutaneous bilirubin (TcB) devices that estimate serum bilirubin noninvasively have been found to reduce the need for blood draws from neonates [7]. The American Academy of Pediatrics recommends the use of TcB devices for the screening of jaundice in infants at more than 35 weeks of gestation [10]. The point of care TcB devices have been in use in the developed countries for a long time. Transcutaneous bilirubinometry was introduced into clinical practice in 1980 by Yamanouchi et al. [11]. The JM103 was first used in 2003 in Japan [12].

In 2004 the American Academy of Paediatrics recommended pre discharge measurement of bilirubin using point of care TcB for babies more than 35 weeks gestational age [13, 14]. These devices, when used prior to the commencement of phototherapy, have been shown to correlate well with TSB levels in both term and preterm infants [6, 15–18]. There are, however, conflicting reports on the use of the JM103 in newborns of African origin. The device has been reported to overestimate bilirubin levels in pigmented African Nigerian and African American newborns [19, 20]. Studies from Malawi indicated that the JM103 could be used to assess jaundiced babies for phototherapy [21]. In Malawi, the JM103 has been used to guide phototherapy in jaundiced newborns [21].

We, therefore, set out to evaluate the accuracy of TcB measurements using the Draeger JM 103 device in assessing jaundice in black Zimbabwean newborns against the diazo method as the gold standard.

## Methods

### Study design

Analytical Cross Sectional Study.

### Study setting

The study was conducted at Harare Central Hospital (Zimbabwe) between 1st August and 30th November 2015. The hospital is the largest teaching and specialist health care center in Zimbabwe. The maternity unit has on average, 1200 deliveries and 400 neonatal admissions per month.

### Study subjects

Hospitalized newborns with visible jaundice, where phototherapy had not yet been commenced.

### Inclusion Criteria

Jaundiced infants aged 0–10 days before commencement of phototherapy.

### Exclusion Criteria

Infants with major congenital abnormalities, severely dehydrated infants with poor peripheral perfusion.

#### Laboratory tests

##### Draeger JM103 assessments

Paired TcB and TSB measurements were performed on eligible infants. TcB was measured on the forehead and on the sternum using a Draeger JM 103 (Medical Systems, USA). Three TcB measurements were done for each site and an average was taken. The TcB machine was calibrated daily according to the manufacturer’s instructions.

#### TSB estimation

A venous blood draw for TSB was done within 30 min of the TcB measurement. TSB was measured in the laboratory by a photometric technique using diazo methods. The hospital laboratory has two types of analyzers, Dimension Xpand Plus (Siemens, Germany) and Mindary BS 400 (China). TSB measurements were performed using either of these machines. The analyzers are always calibrated daily in the morning as part of the standard operating procedure of the laboratory.

The TcB measurements and TSB were entered onto a log sheet alongside with the following variables: gestational age, gender, age of infant and type of treatment given to patient (phototherapy, exchange transfusion or observe). The patients were managed according to WHO guidelines for management of jaundiced newborn.

Permission to conduct the study was obtained from Harare Hospital Ethics Committee Approval number HCHEC 200515/39. Written Informed consent was obtained from the parents.

#### Data management and analysis

Data were entered into EPI INFO Version 6.4 and then exported to STATA version 12 for cleaning and analysis.

The Pearson Correlation Coefficient (r) was calculated for the two TcB measurement sites, forehead and sternum. Linear regression plots of the JM-103 TcB versus TSB estimates were plotted. A comparison was made between the preterm and term babies for the two measured sites. Using the TSB as the gold standard, the specificity and sensitivity of the JM103 TcB measurements were calculated including the positive and negative predictive values. The Bland-Altman plot was used to check for agreement between the TSB and Forehead TcB measures. McNemar’s test was used for sensitivity and specificity comparison between the TcB Sternum and TcB Forehead diagnostic tests. The Receiver Operator Characteristic curves were used to access for the accuracy of the two diagnostic tests.

## Results

Two hundred and eighty-three newborns were recruited during the study period. Fifty-five percent (55%) were male. The gestational age ranged from 28 to 42 weeks with a median gestational age of 38 weeks [Q1, 34; Q3, 40]. Forty-one percent (41%) were preterm. The median postnatal age was 3 days [Q1,3; Q3, 4, range 1–10 days]. Table 1 shows the clinical characteristics of the study participants.

**Table 1:** Clinical characteristics of the study participants

Characteristic		Number (*n*)	Percentage (%)
Gender	Male	155	55
	Female	127	45
Gestational age (weeks)	28–36	115	41
	≥37–42	168	59
Postnatal age at measurement	≤24 h	9	3
	2 days	54	19
	3 days	84	30
	4 days	73	25
	5–10 days	58	21

**Fig. 1:**
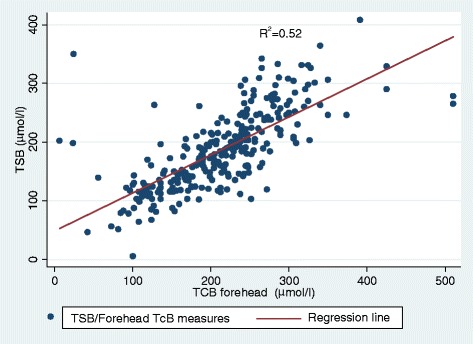
Linear Regression Plot for Forehead TcB Versus TSB. Linear regression plot showing the relationship between Forehead TcB and TSB. As Forehead TcB concentration increase TSB concentration also increase showing a positive linear relationship with a coefficient of determination, R^2^ = 0.52

**Fig. 2:**
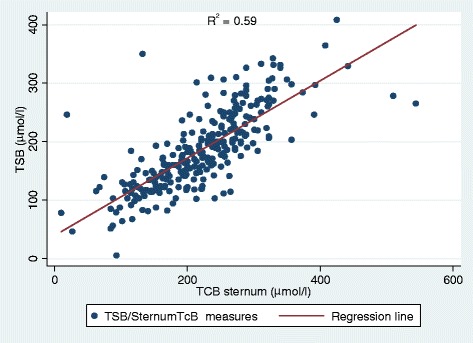
Linear Regression Plot of Sternum TcB versus TSB. Linear regression plot showing the relationship between Sternum TcB and TSB. As Sternum TcB concentration increase TSB concentration also increase showing a positive linear relationship with a coefficient of determination, R^2^ = 0.59

TSB ranged between 85 and 408 μmol/l. Sternum TcB ranged between 170 and 544 μmol/l and forehead TcB 119–510 μmol/l as shown in Table 2. The correlation between paired serum bilirubin and sternum TcB was 0.77, and that between the TSB and forehead TcB was 0,72 as shown in Table 3 and Figs 1 and 2. The paired correlation in preterm babies was 0.77 for sternum and 0.75 for forehead. The correlation was 0.76 for sternum and 0.70 for forehead for term babies as shown in Table 4.

**Table 2:** Summary statistics (TSB mean, TcB mean, median)

Variable	Number of patients	Minimum value	Max	Mean	Std Deviation
TSB	283	85	408	186	66,1
TcB Sternum	283	170	544	221	76,7
TcB forehead	283	119	510	212	73,8

**Table 3:** Pearson correlation coefficient (r) of TcB sternum and TcB forehead

Variable	r	Confidence Interval	*p*-value
TcB forehead	0.724	0.663–0.775	<0,001
TcB Sternum	0.766	0.714–0.811	<0,001

**Table 4:** Pearson Correlation coefficient (r) of TcB sternum and TcB forehead for term and preterm babies

TcB site	Gestational age	r
Forehead	28–36 weeks	0.7548
	≥37 weeks	0.7048
Sternum	28–36 weeks	0.7691
	≥37 weeks	0.7614

Using cut offs for phototherapy according to the WHO guidelines, the sensitivity of the TcB sternum was 76% [95% CI 64–86] and specificity was 90% [95% CI 86–94]. The Positive Predictive Value (PPV) was 70 [90% CI 58–81] and Negative Predictive Value (NPV) was 92 [95% CI 88–96]. The sensitivity for the TcB (forehead) was 62% [95% CI 52–77] and specificity 95% [95% CI 91–97] with a PPV of 80 [95% CI 67–89] and NPV of 90 [95% CI 85–93]. There was no difference in the sensitivity (*p* = 0.1799) and the specificity (*p* = 0.0648) for the two anatomical sites.

**Fig. 3:**
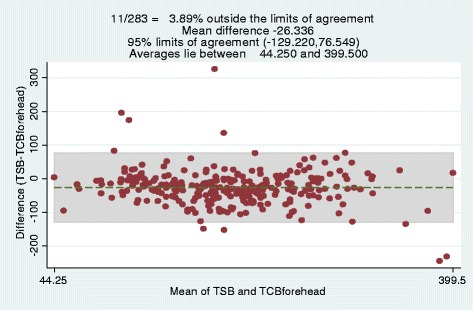
Bland Altman Plot showing difference between TSB and TcB forehead. The Bland Altman plot shows the agreement between Forehead TcB and TSB measurements. The plot shows that only 3.89% of the points lie outside the limits of agreement

Bland-Altman plot of TSB versus TcB forehead showed agreement between the tests. Only 11/283 (3,89%) of the tests were outside the limits of agreement with a mean difference of −26.34 as shown in Fig. 3.

### Receiver operator characteristic curves

**Fig. 4:**
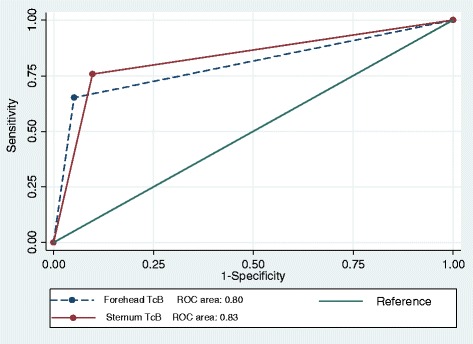
Receiver Operator Characteristic Curves for Sternum and Forehead TcB. The ROC curve shows the accuracy of Forehead TcB and Sternum TcB. The ROC curves show good accuracy in the two methods in separating babies needing phototherapy and those not in need with no significant difference in the two methods

The accuracy of the two two diagnostic tests were compared using the ROC curves as shown in Fig. 4. The area under the Forehead TcB curve = 0.80 and the area under the Sternum TcB curve = 0.83. The difference was not statistically significant though, *p* = 0.2954. Given the area under the curve for each test, the two anatomical sites can be classified as good at separating babies needing phototherapy and those not in need.

## Discussion

The study was performed on newborns in a tertiary neonatal unit of a resource constrained setting. The gestational age ranged from 28 to 42 weeks. There was agreement between transcutaneous bilirubin values and serum bilirubin levels for both sternum and forehead. The study demonstrated a strong correlation between TcB (both sternum and forehead) and TSB in an ethnic Zimbabwean population. There are a number of previous reports of validation of the Draeger JM103 machine in different populations [6, 15, 16, 18, 19, 21]. In this study, the site of TcB measurement and gestational age were examined. Other studies have examined the postnatal age, severity of illness of the infant, effect of phototherapy on TcB and type of instrument used to measure the TcB [16, 19, 21, 22].

There are huge variations in the number of patients and inclusion criteria in the studies [17–19, 21]. Some studies excluded premature infants less than 35 weeks gestation and sick infants [23]. Our study included sick newborns and included gestational ages 28 to 42 weeks. Several researchers have shown that race has a significant effect on TcB with Caucasian infants having higher TcB values than nonwhite infants for similar TSB concentrations [19, 23, 24]. The infants in our study were all black Zimbabweans.

The forehead is the most frequent site for TcB measurement in clinical practice. In our study, the correlation coefficient for TcB (sternum) was higher than TcB (forehead). However, the difference was not statistically significant. Various studies have shown similar results with better correlation of TSB to TcB (sternum) compared to any other part of the body and TcB (forehead) more likely to under estimate TSB compared to TcB (sternum) as has been found in other studies [6, 19, 22, 23, 25]. The reason for this difference between sternum TcB and forehead TcB is not clear. It has been postulated that the difference could be because the sternum is usually covered whereas the fore head is usually exposed to sunlight ultraviolet rays. Exposure to ultraviolet rays stimulates melanin production. This results in reduction of the basal yellow skin colour, which TcB devices measure [26]. In some studies, gestational age, as well as postnatal age, have been shown to have an effect on TcB. This could be attributed to maturation of skin seen in term babies and changes in albumin binding which occurs with maturity [26]. In the current study, there was a better estimation of TSB by TcB for both anatomical sites in the less than 37 weeks gestation compared to those more than 37 weeks though this was not statistically different [26]. There were very few preterms in the current study and this could probably explain why there was no statistical difference in the correlation coefficients between the term and premature newborns.

There are conflicting reports on the effect of TCB at high serum bilirubin levels. Some reports suggest that TcB results may become less accurate with a tendency to overestimate TSB at high bilirubin concentrations [17, 27, 28]. Olusanya O. B. reported overestimation of jaundice using the JM 103 TcB machine on Nigerian newborns [20]. Other studies have found a tendency of TcB to under estimate serum bilirubin levels at high concentrations [6, 25, 29]. In the current study, we did not explore the effect of TcB on high concentrations of bilirubin. Generally, normo grams in use are based on total serum bilirubin levels. The gold standard Laboratory methods for measuring bilirubin are not without their own inherent limitations. Rubattelli et al. reported that standard methods of evaluating TSB tended to under estimate serum bilirubin at higher concentrations [30].

### Limitations of the study

The study was done on ill newborn babies. We are not sure if the results are reproducible on well babies with jaundice in the outpatient set up.

The gestational age range was wide (28–42 weeks). There might be variations according to gestation. We did not have enough numbers to use narrow gestational age ranges for comparison.

## Conclusion

This study established the validity of the Draeger JM103 transcutaneous bilirubinometer in indigenous Zimbabwean newborns. In this population, this noninvasive method of measuring jaundice may be considered as a screening tool for babies who require serum bilirubin and possibly decision to commence phototherapy. Our data support the use of sternum TcB in this population because it had less false negatives. Use of the TcB devices is recommended for decreasing blood sampling during screening for neonatal jaundice. There is a need, however, to establish local nomograms for the cutoff thresholds for treatment based on TcB rather than TSB levels. Further studies are recommended to explore the effect of high serum bilirubin levels on TcB.

## Abbreviations

ABE: Acute Bilirubin Encephalopathy
Draeger JM 103: Draeger Jaundice Meter 103
NPV: Negative predictive value
PPV: Positive predictive value
TcB: Transcutaneous bilirubin
TSB: Total serum bilirubin
